# Anti-UVC Irradiation and Metal Chelation Properties of 6-Benzoyl-5,7-dihydroxy-4-phenyl-chromen-2-one: An Implications for Anti-Cataract Agent

**DOI:** 10.3390/ijms12107059

**Published:** 2011-10-21

**Authors:** Jiahn-Haur Liao, Tzu-Hua Wu, Feng-Lin Hsu, Yi-Shiang Huang, Po-Hung Chiang, Zih-You Huang, Chi-Hsien Huang, Shih-Hsiung Wu, Mei-Hsiang Lin

**Affiliations:** 1 Institute of Biological Chemistry, Academia Sinica, 128 Academia Road, Section 2, Nankang, Taipei 115, Taiwan; E-Mails: liaojh@gate.sinica.edu.tw (J.-H.L.); pokerface1031@gmail.com (Y.-S.H.); 2 School of Pharmacy, College of Pharmacy, Taipei Medical University, 250 Wuxing Street, Taipei 11031, Taiwan; E-Mails: thwu@tmu.edu.tw (T.-H.W.); hsu0320@tmu.edu.tw (F.-L.H.); bobjbh@hotmail.com (P.-H.C.); b3o3o97110@tmu.edu.tw (C.-H.H.); 3 Institute of Biochemical Sciences, National Taiwan University, No. 1, Section 4, Roosevelt Road, Taipei 10617, Taiwan; E-Mail: stanleyhzy@gmail.com

**Keywords:** irradiation, anti-cataract, coumarin, γ-crystallin, iron-chelation

## Abstract

Coumarin derivative 1, 5,7-dihydroxy-6-(3-methyl-1-butyryl)-4-phenyl-chromen- 2-one, has been reported to possess radical scavenging activity and DNA protection. We have synthesized a series of coumarins with structural modifications at positions C4, C5, C6 and C7 and evaluated them for their anti-UVC properties. Coumarin 7, 6-benzoyl-5,6-dihydroxy-4-phenyl-chromen-2-one, was found to have the most potent activity in protecting porcine γ-crystallin against UVC insults. Results of fluorescence assays indicated that compound 7 was capable of decreasing the loss of intensity while lens crystallins and DNA PUC19 were irradiated with UVC. Presence of compound 7 decreased hydroxyl radical levels determined by probe 1b and the free iron concentrations determined by Ferrozine reagent. The chelation assay showed that compound 7 was chelated to metal via 6-CO and 5-OH on the benzopyrone ring. The observed protective effects of compound 7 towards crystallins from insults of UVC and free radicals may be due to its iron-chelating activity and its peak absorption at 254 nm.

## 1. Introduction

Solar radiation can be divided into five regions in increasing order of wavelengths: ultraviolet UVC, UVB, UVA, visible range, and infrared range. Among these radiations, UVC and UVB are responsible for photochemical reactions. UVA, visible-range, and infrared-range radiation were traditionally thought to be less damaging. UV-induced oxidative damage is highly related to cataractogenesis [1,2]. Compounds with anti-UV properties can be used as anti-cataract agents. Coumarin derivatives were reported to protect rabbit corneal-derived cells (SIRCs) from UVB-induced oxidative damage [3].

Coumarins are a class of phenolic substances commonly found in plants, fungi, and bacteria [4]. Structures of these compounds consist of a benzene moiety fused to an α-pyrone. The structural diversity found in this family of compounds can be classified into different categories, from coumarins to polycyclic coumarins, such as furocoumarins and pyranocoumarins. This diversity leads to multiple pharmacological activities; natural and synthetic coumarins have anti-inflammatory [5,6], antimicrobial [6,7], antiviral [6,8], anticarcinogenic [6,9], anticoagulant [6,10], and antioxidant activities [6,10]. Generally speaking, their mechanisms of action are not well understood.

Among activities of coumarin derivatives, their antioxidant properties have garnered attention due to their potential applications. Compounds containing the moiety of coumarin (1,2-benzopyrone, 2*H*-1-benzopyran-2-one, 2) were shown to possess antioxidant activity [11–20]. Many different types of antioxidant activity of coumarins were investigated in various biological systems, but it was difficult to determine a structure-activity relationship [5,6,11,21]. The scavenging capacities of coumarins against various free-radical types were investigated. Those results implied that compounds with phenolic hydroxyl groups are thought to possess antioxidant activities. A hydroxyl group on an aromatic ring structure can carry out the single-electron reduction of a free radical; therefore, compounds with phenolic hydroxyl groups are good radical scavengers.

The eyes are constantly exposed to reactive oxygen species (ROS) induced by UV light [22]. A previous report showed that corneal and tear fluids possess an elaborate antioxidant defense system [23]. However, UV light can pass through the cornea and enter the lens. UV irradiation is one of the significant risk factors in cataractogenesis. Chemical modifications or oxidation of lens proteins may also contribute to the emergence of opacities. Eye drops containing antioxidants such as tropicamide, phenylephrine, flurbiprofen, and tobramycin are also given preoperatively [24]. In recent years, the protective effects of compounds against UV-induced oxidative damage of the eye were investigated [3,25,26]. It was shown that UV irradiation can cause turbidity of γ-crystallin [27,28]. Although the detailed mechanism of this phenomenon remains vague, it is believed to be related to ROS induced by UV light. The focus on the antioxidant activities of coumarin derivatives brought our attention to cataracts, which are thought to be correlated with oxidative stress [29–34].

**Chart 1:**
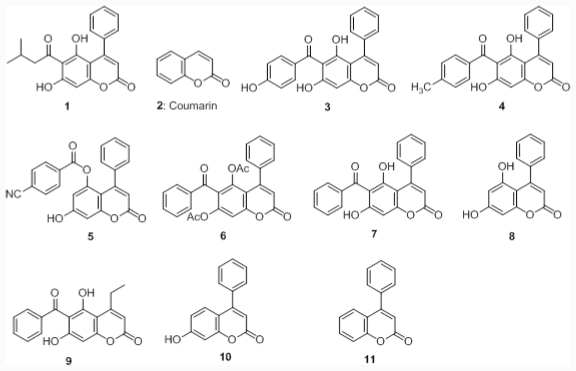
Structures of coumarin derivatives.

Previously, we showed that coumarin 1 (Chart 1) possess anti-inflammatory, antioxidant activities, and the pUC-19 plasmids co-incubated in the presence of coumarin 1 exhibited less DNA strand damage and most of the plasmids were in the supercoiled form [20]. Those results demonstrated that our 6-acyl-4-aryl-alkyl-5,7-dihydroxycoumarins have the ability to scavenge the hydroxyl radical under Fenton reaction conditions. Since phenolic compounds are well-known antioxidants, we are interested in examining the antioxidative effects of the compounds where (i) a phenolic moiety is attached to the 6-acyl position of 6-acyl-4-phenyl-5,7-dihydroxycoumarin; (ii) the hydroxyl group of 6-benzoyl is replaced with methyl or hydrogen; and (iii) 5- and 7-hyroxyl groups are present on the coumarin ring. Therefore, here we aimed to established an *in vitro* anti-UVC assay and use it to screen for the most potent anti-UVC radiation compound from the library of synthetic coumarin derivatives 3**–**11 (Chart 1). This compound was further investigated towards crystallins and DNA protection, and the involvement of iron-chelation activity was then further examined by Ferrozine assay and NMR spectroscopy.

## 2. Results and Discussion

### 2.1. Chemistry

**Scheme 1:**
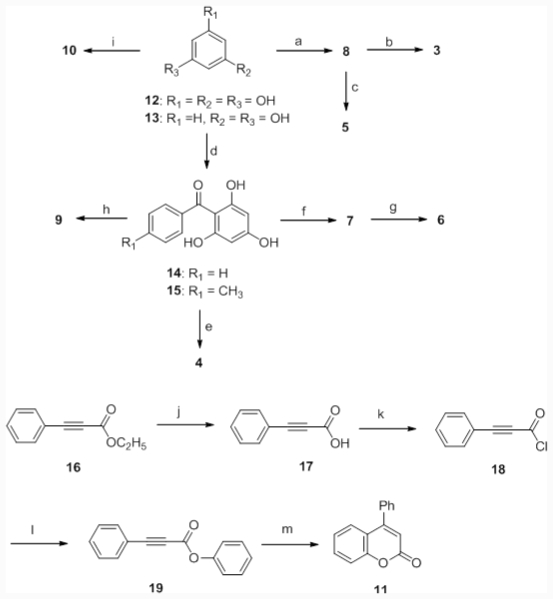
Synthesis of coumarin derivatives. Conditions: **a**: 12, ethyl benzoylacetate, trifluoroacetic acid, reflux, 40%; **b**: *p*-anisoyl chloride, AlCl_3_, nitrobenzene, 60 °C, 3 h, 16%; **c**: *p*-cyanobenzoyl chloride, AlCl_3_, nitrobenzene, 60 °C, 3 h, 15%; **d**: 12, benzoyl chloride, AlCl_3_, nitrobenzene, 60 °C, 3 h, 15%; **e**: 15, *p*-toluoyl chloride, AlCl_3_, nitrobenzene, 60 °C, 3 h, 36%; **f**: 14, ethyl benzoylacetate, glacial HOAc, conc. H_2_SO_4_, 15%; **g**: sodium acetate, acetic anhydride, 160 °C, 1 h, 86%; **h**: 14, ethyl propionylacetate, glacial HOAc, conc. H_2_SO_4_, 27%; **i**: 13, ethyl benzoylacetate, glacial HOAc, conc. H_2_SO_4_, 60%; **j**: 1.5 N NaOH, THF, reflux 1 h, 75%; **k**: oxalyl chloride, THF, DMF; **l**: phenol, NaH, THF; **m**: 5 mol% AuCl_3_, AgOTf, 70%.

Coumarin is a flavonoid, which is a natural constituent of many plants and essential oils. Coumarins from plants are derivatives of cinnamic acid with a benzo-α-pyrone skeleton [35]. They are classically synthesized by the Perkin, Pechmann, or Knoevenagel reactions [6]. Pechmann condensation utilizes a phenol and carbozylic acid or ester for the coumarin synthesis. We synthesized several coumarin derivatives using this method [20,36]. Herein, we followed a similar strategy to synthesize most of coumarin derivatives (Scheme 1).

### 2.2. Biological Results

#### 2.2.1. Turbidity Assay of γ-Crystallin and Comparison of Anti-UVC Activity

**Figure 1:**
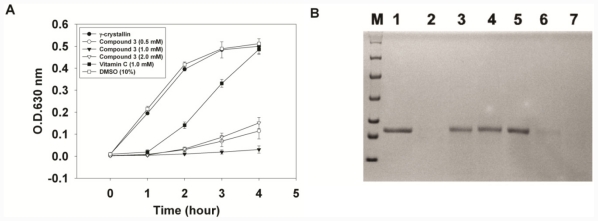
Turbidity assay of compound **3** under UV irradiation. (**A**) Porcine γ-crystallins, with or without compound **3**, were irradiated by UV (254 nm), and the turbidity was measured at 630 nm by ELISA reader. Solvent, DMSO, and ascorbic acid were used as controls. The bars denote ranges of variation from four measurements; (**B**) SDS-PAGE of porcine γ-crystallin before and after UV irradiation (4 h) in various conditions. Lane 1: γ-crystallin before UV irradiation. Lane 2: γ-crystallin after UV irradiation. Lanes 3, 4, and 5: γ-crystallin with compound 3 (0.5, 1.0, and 2.0 mM, respectively) after UV irradiation. Lane 6: γ-crystallin with ascorbic acid (1.0 mM) after UV irradiation. Lane 7: γ-crystallin with DMSO (10%) after UV irradiation. M: markers. The masses of markers are 116.0 kDa, 66.2 kDa, 45.0 kDa, 35.0 kDa, 25.0 kDa, 18.4 kDa and 14.4 kDa from the top to bottom of the gel.

Previously, UVC irradiation to induce turbidity of γ-crystallin was applied to measure the chaperone activity of lens γ-crystallin [24,25]. Lens γ-crystallin was correlated to cataracts and demonstrated in a mouse model [37]. Formation of amyloid fibrils *in vitro* from partially unfolded intermediates of human γ-crystallin was investigated [38]. Amongst the lens crystallins, γ-crystallins are particularly sensitive to oxidation, because of their high amounts of Cys and Met residues [39]. Therefore, we chose γ-crystallins as the reported proteins for our assays. Compounds that protect γ-crystallins from damage by UVC irradiation can be anti-cataract agents. Hydroxycoumarins were previously shown to have antioxidant activities [11,20,21]. They are believed to act like classic phenol- or quinol-based antioxidants which can carry out the single-electron reduction of a free radical. Compound 3 (5,6-dihydroxy-6-(4-hydroxybenzoyl)-4-phenyl-2*H*-chromen-2-one) was dissolved in DMSO to create a stock solution which was then added to the sample solution to achieve a maximum final DMSO concentration of 10% (v/v). The sample solutions of 200 μL each were exposed to a UV lamp (254 nm), and turbidity was measured before and after UV irradiation. For each condition, four sample solutions were used in the experiment. The results indicated that compound 3 can effectively suppress the turbidity induced by UV irradiation (Figure 1A). After UV irradiation, the sample with 10% DMSO showed a slight increase in turbidity, whereas the sample with vitamin C (1 mM) showed a delayed increase of turbidity. It was obvious that vitamin C possesses statistically significant less anti-UV activity than compound 3 at 4 h observation (*P* < 0.05). SDS-PAGE analysis showed that γ-crystallin was effectively protected by compound 3 (Figure 1B), whereas the samples with vitamin C and DMSO only (10%) were photo-degraded after 4 h of UV irradiation.

In order to study the structural activity relationship, various compounds were compared in the same activity assay (Table 1). Due to differences in the solubilities and colors of these compounds, the change in turbidity was used in this assay. Porcine γ-crystallins with or without compounds were irradiated by UVC (254 nm), and the turbidity was measured at 630 nm on an ELISA reader. The changes in turbidity after 4 h of UVC irradiation were recorded, and a sample of only porcine γ-crystallin, without other compounds, was used as a blank. The results indicated that potency ranking of anti-UVC activity was as follows: 7 > 4 = 6 > 3 > 9 > 10 > 11 > 8 > 2 > 5 (Table 1). Coumarin (2) showed 20% suppression of turbidity, and SDS-PAGE showed it had poor protective activity. Compounds 3–7 respectively showed 66%, 103%, 12.5%, 99.9%, and 116% suppression of turbidity. SDS-PAGE also indicated that compounds 3, 4, 6, and 7 showed good protective activities. Compound 3 possesses three phenolic hydroxyl groups, but it showed only 66% suppression of turbidity. Compound 4 was more potent than compound 3 indicating that the 6-*p*-methylbenzoyl group is more potent than the 6-*p*-hydroxybenzoyl group. Compound 7 showed the best protective activity among them. The 6-*p*-hydroxybenzoyl group was less potent than the 6-benzoyl group when comparing compounds 3 and 7. Compound 5 showed only 12.5% suppression of turbidity, and SDS-PAGE showed that it had less protective activity, which indicated that the 5-hydroxyl group may play an important role in protective activity. Substitution of the 5,7-dihydroxyl groups in compound 7 with *O*-acetyl groups (compound 6) did not show significant effects in augmenting protective activity. However, compound 6 showed less solubility in the buffer. The result indicated that compound 7 was the most potent compound in this study.

Previous reports have indicated that water irradiated by UVC forms free radicals [40,41]. It was reported that ascorbic acid exhibits potent free radical-scavenging activity and was used as the control [42]. Ascorbyl radicals are long-lived free radicals which make vitamin C a good radical scavenger [43]. The sample with vitamin C (1 mM) showed a delayed increment in turbidity after UVC irradiation. A high concentration of vitamin C can protect γ-crystallin from photodegradation by UVC. Hence, photodegradation of γ-crystallin can be correlated to free radical reactions.

#### 2.2.2. Evaluation of the Substitution Groups

An additional interesting aim of the investigation was to determine the structure-activity relationship of coumarin derivatives. From the above study, it was shown that substitution of a 6-benzoyl group in compound 7 affected its activity. Therefore, by means of the Pechmann reaction, we synthesized compound 8 which does not contain a 6-benzoyl group (Scheme 1). The anti-UVC activity assay showed that compound 7 was more potent than compound 8. Compound 7 showed 94% suppression of turbidity, whereas compound 8 showed only 36% suppression of turbidity. SDS-PAGE showed that both compounds could protect against γ-crystallin degradation (data not shown). Hence, we concluded that the 6-benzoyl group promoted anti-UVC activity. This result may have been due to the addition of the 6-benzoyl group helping to stabilize free radicals at position 6 when phenolic hydroxyl groups carry out the single-electron reduction.

To evaluate the function of the 4-phenyl group of compound 7, compound 9 was synthesized in a similar way as compound 8 (Scheme 1). The 4-phenyl group was changed to a 4-ethyl group in compound 9. The results of the activity assay revealed that compound 7 showed 89.3% suppression of turbidity, whereas compound 9 showed only 54% suppression of turbidity. It was demonstrated that compound 7 was more potent than compound 9. SDS-PAGE showed that both compounds can protect against γ-crystallin degradation (data not shown). The 4-phenyl group also played a role in promoting the anti-UVC activity of compound 7. It was proposed that the addition of a 4-phenyl group would enhance the resonance effects of coumarin derivatives.

Table 1 shows the comparisons of all tested coumarin derivatives. Results show that coumarin possesses poor anti-UVC activity. The addition of hydroxyl groups to coumarin derivatives greatly improved the anti-UVC activities of these compounds. Compound 5 showed poor anti-UVC activity, indicating that the 5-hydroxyl group may play an important role in the activity of compound 7. Therefore, we synthesized compounds 10 and 11 (Scheme 1) to investigate the influence of the phenolic hydroxyl group. The anti-UVC activity assay of compounds 8, 10, and 11 demonstrated that both compound 10 and 11 possessed poor activity. Compound 8 showed 77.8% suppression of turbidity, while compounds 10 and 11 respectively showed only 68.7% and 35.8% suppression. The SDS-PAGE analysis also revealed that compounds 10 and 11 possessed poorer protective activities (data not shown). Compound 11 exhibited slightly higher anti-UVC activity than coumarin, which indicates that the 5,7-dihydroxyl groups are important in this activity assay. However, compared to compound 11, the activity of compound 10 was lower by about 10%, but SDS-PAGE showed that γ-crystallin was severely degraded. Comparing compounds 10 and 5 showed that the 5-*p*-cyanobenzoyl group of compound 5 led to the poor activity. The phenolic hydroxyl group improved the anti-UVC activity of the compound. It should be noted that hydroxyl groups also improved the solubility of the coumarin derivatives in the water-based buffer.

#### 2.2.3. Protection of DNA

**Figure 2:**
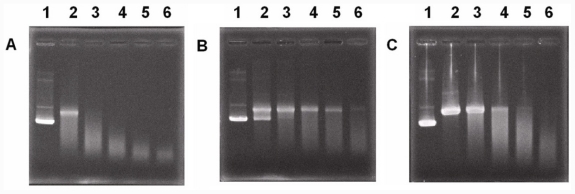
Activities of compound 7 and vitamin C in protecting DNA from photodegradation by UV. (**A**) Plasmid DNA (PUC19) was broken and then degraded under UV irradiation. Lane 1, supercoiled DNA without UV irradiation; lanes 2–6, DNA after UV irradiation for 30, 60, 90, 120, and 150 min, respectively; (**B**) Plasmid DNA incubated with compound 7 (1 mM) showed that some fraction of DNA was preserved under UV irradiation; (**C**) Plasmid DNA incubated with vitamin C (1 mM) showed that most DNA became the linear form and then degraded under UV irradiation.

It was demonstrated that compound 7 possessed the best anti-UVC activity among these coumarin derivatives. However, UVC irradiation damages both proteins and DNA. UVC irradiation may damage the DNA of cells. The outside surface of the lens is a layer of lens epithelial cells. UVC damage to lens epithelial cells may lead to acute UVC cataractogenesis [44]. Therefore, the ability of compound 7 to protect DNA from UVC damage was tested (Figure 2). Supercoiled DNA (PUC19 plasmid) irradiated by UVC (254 nm) became linear DNA and then photodegraded. The addition of compound 7 prevented the supercoiled DNA from photodegradation. After 30 min of irradiation, a portion of the supercoiled DNA was preserved in the presence of compound 7, while all DNA had become linear in the presence of vitamin C. It was shown that the UVC-protective property of compound 7 is more potent than that of vitamin C.

#### 2.2.4. Compound 7 Protects Porcine Lens Proteins from Oxidative Damage by Hydroxyl Radicals

**Figure 3:**
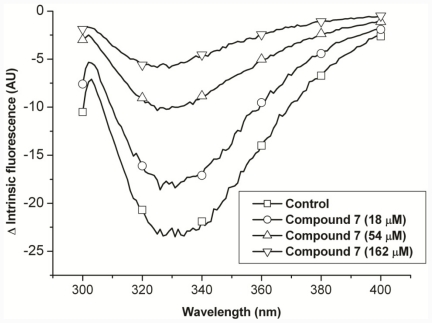
Free radical-scavenging activity. Porcine crystallins were subjected to oxidative stress by Fenton solutions with/without the protection of compound 7. Negative changes indicate the loss of fluorescence intensity of tryptophan of lens crystallins. The protective activity of compound 7 was concentration-dependent.

In the abovementioned discussion on the mechanism of compound 7’s anti-UVC activity, we have assumed that compound 7 possesses free radical-scavenging properties. Therefore, we tested if compound 7 can protect lens crystallins from the Fenton reaction (Figure 2). Although all amino acid residues in the protein chain are susceptible to modification by the hydroxyl radical, tryptophan is the most vulnerable amino acid. In our previous report, the intrinsic fluorescence of lens crystallins was used as an index of the effectiveness of the tested compound [45]. Herein, we showed that compound 7 protected lens crystallins in a dose-dependent manner (Figure 3). The solvent, DMSO, had no protective function. It was demonstrated that compound 7 is a free radical scavenger and can protect crystallins. However, the commonality between UV irradiation and the Fenton reaction was not provided. It is known that hydroxyl radicals are produced during UV-light dissociation of H_2_O_2_. Hence, we tested if UV irradiation increased H_2_O_2_ in solution. A sensitive probe for H_2_O_2_ was used to evaluate H_2_O_2_ produced by UV irradiation [46]. These results indicated that UV irradiation produced H_2_O_2_ and showed some commonality with the Fenton reaction (data not shown).

### 2.3. Iron-Chelating Activity

**Figure 4:**
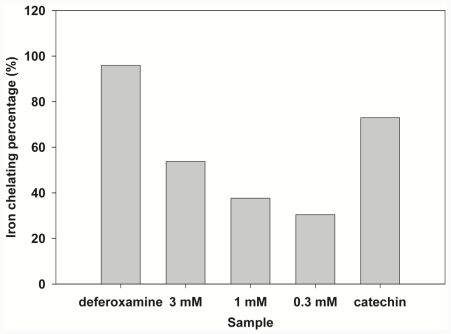
Iron-chelating activity of compound 7. Comparative analyses of ferrous ion-chelating potency with various concentrations of compound 7, and positive controls (30 mM of catechin or deferoxamine) were performed to determine their ability to complex with ions. The percentage of the chelating effect was calculated using the following equation: [1 – (absorbance of a sample at 562 nm)/(absorbance of the control, without tested a sample or positive control, at 562 nm)]/100.

**Figure 5:**
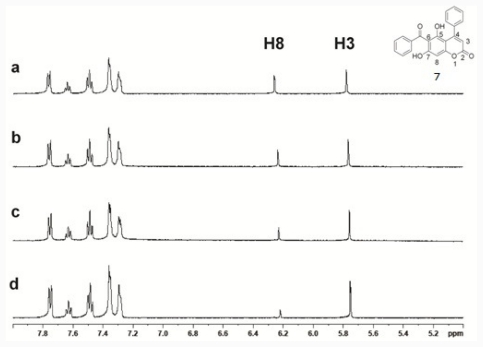
1D-nuclear magnetic resistance (NMR) spectrum of 5 mM of compound 7 without and in the presence of ZnAc in 50 mM Tris-HCl at pH 7.2 (50/50, v/v, DMSO-d_6_/D_2_O-Tris-HCl): (**a**) at a 1: 0; (**b**) at a 1: 0.5; (**c**) at a 1: 1; and (**d**) at a 1: 2 Zn equivalent.

Although compound 7 with phenolic hydroxyl groups showed free radical-scavenging properties, there are other possibilities in preventing the Fenton reaction. A previous report indicated that the chelator is capable of binding ferrous and ferric iron, reducing concentrations of Fe(II) and Fe(III) in solution, and thus alleviating the Fenton reaction [47]. In view of its chemical structure, results suggested that compound 7 can chelate Fe(II). The chelation assay showed that compound 7 was capable of binding ferrous iron (Figure 4). To further confirm this property, ^1^H NMR titration studies were performed to identify the likely site of iron binding on compound 7. A diamagnetic metal ion, Zn^2+^, was used as the probe for Fe^2+^ [48]. Figure 5 shows the 1D-NMR spectrum of compound 7 in the presence and absence of ZnAc. As Zn^2+^ was added, little change was observed in most of the compound 7 peaks; however, the H^8^ proton peak showed a slight upfield shift (of ca. 0.03 ppm). H^8^ resonance (shifted and broadened) was observed by 1:2 stoichiometric Zn complexation indicating that Zn is chelated to 6-CO and 5-OH on the benzopyrone ring. Since the H^8^ proton experienced a small change of an upfield shift, it is unlikely to have been chelated to 6-CO and 7-OH on the benzopyrone ring. Due to the similar coordination properties of Fe^2+^ and Zn^2+^, it is reasonable to suggest that Fe^2+^ was chelated at the same site as Zn^2+^.

## 3. Experimental Section

### 3.1. General Experimental Conditions

Melting points were taken in a capillary tube using a MEL-TEMP II melting-point apparatus by Laboratory Devices (CA, USA). Nuclear magnetic resonance (NMR) spectra were recorded on a Bruker DMX-500 FT-NMR spectrometer (Madison, WI, USA); chemical shifts were recorded in parts per million downfield from Me_4_Si. IR spectra were determined with a Perkin-Elmer 1760-X FT-IR spectrometer (Massachusetts, USA). Mass spectra were recorded on Jeol JMS-D300 (Tokyo, Japan) and FINNIGAN TSQ-46C mass spectrometers (Thermo scientific, USA); HRMS was obtained with a Jeol JMS-HX110 spectrometer. TLC was performed on Merck (Art. 5715) silica gel plates (Darmstadt, Germany) and visualized under UVC light (254 nm). Flash column chromatography was performed with Merck (Art. 9385) 40–63 μm silica gel 60.

### 3.2. Preparation of Coumarin Derivatives 3–11

Synthesis of coumarins 3–5, 7, and 9 were reported by our laboratory [20,36]. Acetylation of the phenolic hydroxyl group of coumarin derivative 7 was yielded coumarin derivative 6. Resorcinol treated with ethyl benzoylacetate resulted in coumarin derivative 10. Compounds 8 and 11 were prepared by a method reported in the literature [49,50].

### 3.3. Iron-Chelating Activity

The chelation of ferrous ions by compound 7 was estimated using a modified method of Lopes *et al*. [51,52]. Ferrous ammonium sulfate was added to a solution containing 5% ammonium buffer and different concentrations of compound 7 (0.3, 1, and 3 mM). The final concentration of ferrous ammonium sulfate was 0.5 mM. Ferrozine was added to initiate the reaction, and the final concentration was 0.25 mM. The reaction mixture was incubated at room temperature for 15 min after being shaken. The absorbance of the solution was measured at 562 nm in a Helius Alpha spectrophotometer (Unicam Instruments, Cambridge, UK). Deferoxamine was used as a control at a final concentration 30 μM. The higher the iron-chelating activity of a tested sample was, the lower the resulting ultraviolet absorbance. Therefore, the relative iron-chelating capacity was calculated as the percent decrease in absorbance of the test samples compared to the controls that lacked the test drugs.

### 3.4. ^1^H-NMR Studies of Metal Chelation

^1^H NMR studies were carried out on a Bruker AC 400 spectrometer. A stock solution of 7 (150 mM) was freshly prepared in DMSO-d_6_. Zinc acetate (300 mM) was prepared in D_2_O. Titrations were done in a 50/50 (v/v) DMSO-d_6_/D_2_O solvent buffered with 50 mM Tris-HCl at pH 7.20. The pH of the solutions was determined with a Corning pH meter (Corning, NY, USA) using a Sigma-Aldrich micro combination electrode (St. Louis, MO, USA). The pH meter readings for D_2_O were recorded as pH values, *i.e.*, uncorrected for the effect of deuterium.

### 3.5. Absorption Spectra of Coumarin Derivatives

Coumarin derivatives 2–7 (0.1 mM) were prepared in methanol, and absorption spectra were measured on a UV/Vis Scanning Spectrophotometer (DU series 700, Beckman Coulter, CA, USA).

### 3.6. Biological Assay

#### 3.6.1. Purification of Porcine γ-Crystallin

Porcine lenses were decapsulated and homogenized in buffer containing 50 mM Tris-HCl, 0.1 M NaCl, 5 mM EDTA, 0.01% β-mercaptoethanol, and 0.02% sodium azide (pH 8.0). After centrifugation at 16,060× *g* for 30 min, the supernatant was collected. The supernatant was applied to a TSK HW-55(F) column (Tosoh Bioscience, Japan) and eluted at 25 mL/h. Five well-resolved peaks were obtained and identified as HMα-, α-, βH-, βL-, and γ-crystallins (Data not shown) based on their subunit compositions as revealed by sodium dodecylsulfate polyacrylamide gel electrophoresis (SDS-PAGE) [28,53,54]. Fractions of porcine γ-crystallin were collected, and the protein concentration was determined according to the Bradford method (BioRad Laboratories, USA).

#### 3.6.2. Turbidity Assays of γ-Crystallin under UVC Irradiation

Photoaggregation of γ-crystallin (20–25 μM) was monitored in a buffer of 50 mM Tris-HCl and 0.1 M NaCl at pH 8.0, in the absence or presence of coumarin derivatives. Sample solutions of 200 μL each were exposed to a UV lamp (254 nm) of Ultraviolet Crosslinker CL-1000 (Bulletin UVP-AB-114, USA). Turbidity changes represented by relative light-scattering intensities after irradiation for four hours were measured at 630 nm by an enzyme-linked immunosorbent assay (ELISA) reader. Four sample solutions were used in the experiment for each condition. The percentage of suppression, (Iγ–Ic)/Iγ × 100%, was used to evaluate the anti-UVC activity, where Iγ is the intensity of scattered light for γ-crystallin without a compound, and Ic is the intensity of scattered light for γ-crystallin with a compound. SDS-PAGE (with 5% stacking and 12% resolving gels) was performed as previously reported [55]. Each well contained approximately 4 μg of porcine γ-crystallin.

#### 3.6.3. Protection of Supercoiled DNA from Strand Breakage by UVC Irradiation

The activity of preventing photodegradation was evaluated by incubating compounds with DNA and irradiating them under UV light. Plasmid DNA (PUC 19) was incubated at 25 °C in water, with or without compound 6 or vitamin C. Sample solutions of 200 μL (0.0225 μg/μL PUC19) were each exposed to a UV lamp (254 nm) of Ultraviolet Crosslinker CL-1000 (Bulletin UVP-AB-114, USA), and samples were collected every 30 min. DNA samples were analyzed on 0.8% agarose gels. Each well contained approximately 450 μg of PUC19.

#### 3.6.4. Fenton Reaction

Crude porcine lens proteins were incubated with various Fenton solutions modified based on the method of Huang *et al*. [56]. The final concentrations of Fe^2+^ and H_2_O_2_ in Fenton solutions of experiments were 0.02 and 0.2 mM, respectively. The fluorescence emission spectra were immediately taken at room temperature and recorded with a Hitachi F-4500 FL fluorescence spectrophotometer excited with 295-nm light. The emission spectra were recorded at 300–400 nm using a light slit of 5 nm for both the excitation and emission modes. Spectra of normal lens proteins were used as baselines for calculating changes in the tryptophan fluorescence intensity.

## 4. Conclusions

**Figure 6:**
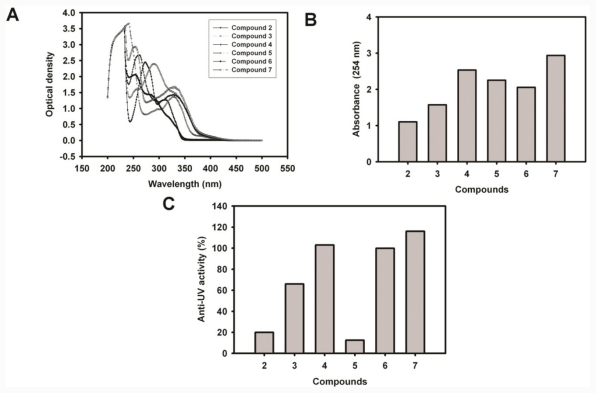
The relationship of UV absorbance of compounds and their anti-UV activity. (**A**) The absorption spectra of compounds 2–7; (**B**) The UV absorbance (254 nm) of compounds 2–7; (**C**) The anti-UV activities of compounds 2–7 are shown in percentages of the suppression of turbidity.

We established an *in vitro* assay to screen compounds with anti-UV properties. Compound 7 was the most potent compound among these coumarin derivatives that suppressed turbidity induced by UV irradiation. The 5,7-dihydroxyl groups play important roles in maintaining the anti-UV activity of this compound. The phenyl group at position 4 and benzoyl group at position 6 of compound 7 are partially responsible for the anti-UV activity. It is reasonable that compounds with a phenolic hydroxyl group possess free radical-scavenging properties. Therefore, coumarin derivatives can protect γ-crystallin and DNA from free-radical damage produced by UVC irradiation. In addition to the free-radical-scavenging properties, we also noted the UV-filtering property of coumarin derivatives. The human lens absorbs most of the UV radiation at 300–400 nm. This absorption is largely performed by a group of low-molecular-weight fluorescent compounds, known as UV-filter compounds [57–59]. In order to evaluate the UV-filtering properties of coumarin derivatives, we measured the UV spectra of these compounds (Figure 6). The optical density at 254 nm of each compound was plotted in this figure. It is interesting that compounds with higher anti-UVC activities possess higher absorption at 254 nm. The only exception was compound **5**, which showed low activity but high absorption. The low activity may have been due to the phenolic hydroxyl group’s inability to carry out the single-electron reduction of the *p-*cyanobenzoyl group, leading to an unstable free radical at position 6. Compound 7 was shown to possess the highest anti-UVC activity and best UV-filtering property at 254 nm. In addition, the iron-chelating activity gives compound 7 an additional potential application in iron-related cataracts. Increasing evidence relating iron to cataractogenesis includes reports of cataract formation following ocular siderosis from an iron foreign body in the lens [60–64]. The benefits of iron chelators are currently being investigated for limiting iron-induced oxidative damage. In conclusion, we have developed a set of *in vitro* assays and 6-benzoyl-5,7-dihydroxy-4-phenyl-chromen-2-one (7) protected lens γ-crystallins against UVC insults and chelated metal via its structure of 6-CO and 5-OH on the benzopyrone ring. Further *in vivo* anti-cataractogenesis assay is warranted.

## Figures and Tables

**Table 1:** Optical density (OD) at 4 h observations. Turbidity assay of various coumarin derivatives under UV irradiation at a concentration of 0.5 mM. Porcine γ-crystallins, with or without co-incubation with test compounds, were irradiated by UV (254 nm) for four hours, and the turbidity was measured at 630 nm with an ELISA reader and expressed as mean ± standard derivation.

**cpd no.**	**2**	**3**	**4**	**5**	**6**
**OD**	0.636 ± 0.041	0.193 ± 0.043	−0.031 ± 0.008	0.698 ± 0.150	0.0003 ± 0.074

**cpd no.**	**7**	**8**	**9**	**10**	**11**
**OD**	−0.041 ± 0.012	0.502 ± 0.012	0.284 ± 0.035	0.364 ± 0.018	0.406 ± 0.032
